# SPRINGing off the lock: the role of SPRING in S1P activity and SREBP-regulated lipid metabolism

**DOI:** 10.1097/MOL.0000000000001003

**Published:** 2025-08-01

**Authors:** Ilaria Micallo, Ashley V. Bullington, Daniel L. Kober, Noam Zelcer

**Affiliations:** a Department of Medical Biochemistry, Amsterdam UMC location AMC, University of Amsterdam; b Amsterdam Gastroenterology, Endocrinology, and Metabolism (AGEM) Institute; c Amsterdam Cardiovascular Sciences (ACS) Institute, Amsterdam UMC, Amsterdam, the Netherlands; d Department of Biochemistry, The University of Texas Southwestern Medical Center, Dallas, Texas, USA

**Keywords:** C12ORF49, cholesterol metabolism, MBTPS1, proteases, scap, site-1-protease, SPRING, SREBP

## Abstract

**Purpose of review:**

Lipid metabolism and de-novo lipogenesis (DNL) is broadly controlled by the SREBP transcription factors. These transcription factors are matured from membrane-anchored precursor proteins by the proteolytic actions of the proteases S1P and S2P. In this review, we summarize the current understanding of SPRING, a recently identified activator of S1P.

**Recent findings:**

Recent studies of SPRING using animal, cellular, biochemical, and biophysical methods have established SPRING as a core component of the SREBP machinery. Deletion of SPRING in cells and animal livers specifically reduces SREBP activity yet leaves other S1P substrates intact, demonstrating an SREBP-specific role for SPRING in licensing S1P activity. Mechanistic biochemical and structural studies revealed that SPRING activates S1P by competitively displacing its inhibitory pro-domain and elucidated how small molecule inhibition of S1P can be accomplished.

**Summary:**

Current studies have shown how SPRING activates S1P and uncovered a critical role for SPRING in the SREBP pathway. Further studies are warranted to understand this emerging, connection between SPRING and the regulation of DNL through SREBP.

## INTRODUCTION AND SCOPE: THE STEROL-RESPONSIVE ELEMENT-BINDING PROTEIN SYSTEM AND DISCOVERY OF THE S1P-SPRING AXIS

**FIGURE 1:**
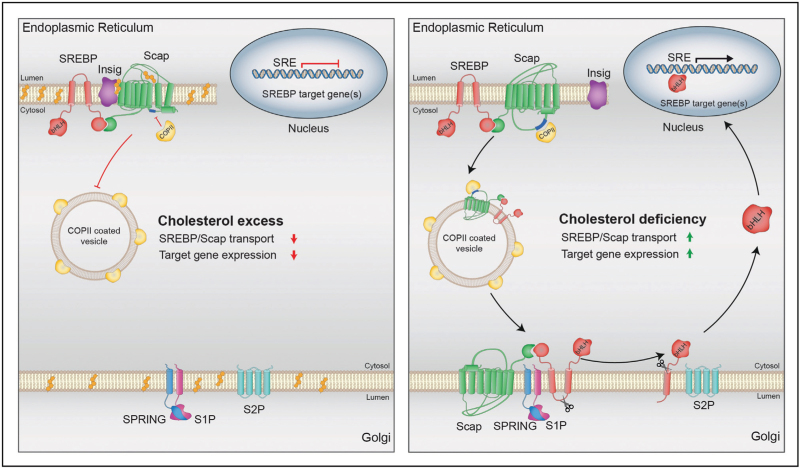
Schematic depiction of the SREBP pathway and its activation. (Left) SREBP and Scap are retained in the ER membrane by INSIG when the ER membrane cholesterol-content is high. This limits transcription of SREBP target genes. (Right) A drop in the cholesterol content of the ER membrane leads to release of INSIG from the SREBP-Scap complex, and to COPII-mediated anterograde transport of the complex to the Golgi. In the Golgi, SREBP undergoes sequential cleavage by S1P, a step licensed by SPRING, and by S2P. The released transcriptionally competent N-terminal domain translocates to the nucleus where it activates the transcription of SRE-containing genes.

Maintaining cellular lipid homeostasis is essential for cell survival and for the ability to adapt to metabolic needs and cues. Herein, the regulation of de-novo lipogenesis (DNL), the topic of this review, plays an important role. DNL is governed by sterol regulatory element binding proteins (SREBPs), a family of transcription factors that guide the transcription of enzymes involved in the synthesis of fatty acids and cholesterol [1]. SREBPs exist in three different isoforms: SREBP1a and SREBP1c, which are transcribed from the same locus but use a distinct transcriptional start site, and SREBP2. These isoforms share high sequence and structure homology but are differentially expressed in distinct tissues and control specific subsets of genes involved in DNL [2,3]. The SREBP transcription factors are produced as large, membrane-bound precursor proteins in the endoplasmic reticulum (ER), where they form a complex with Scap, a cholesterol-sensing protein that harbors a COPII interaction motif. When ER-membrane cholesterol levels are above 5 mol %, the Scap-SREBP complex is retained via its sterol-regulated interaction with Insulin-Regulated Gene (INSIG) (Fig. 1) [1,4–6]. Upon sterol depletion, INSIG releases the Scap-SREBP complex and Scap adopts a conformation that is accessible to the COPII machinery for transport to the Golgi [6,7]. In the Golgi, SREBPs undergo two subsequent cleavages: first by Site-1 Protease (S1P, encoded by *MBTPS1*) in the lumen-facing loop, and then by Site-2 Protease (S2P, encoded by *MBTPS2*) in the intermembrane N-terminal region [5]. The matured SREBP N-terminal domain translocates to the nucleus and induces the transcription of sterol regulatory element (SRE)-containing genes [5,8,9]. 

**Box 1:**
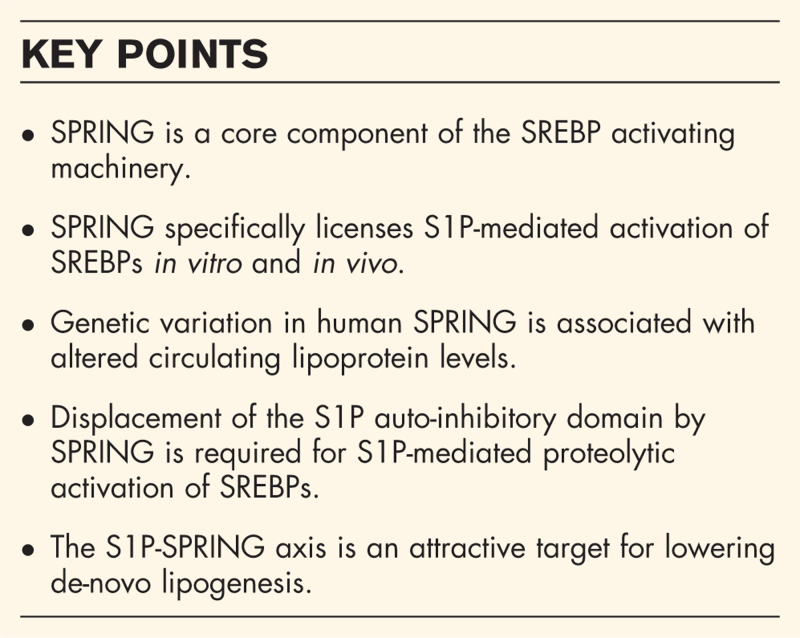
no caption available

**FIGURE 2:**
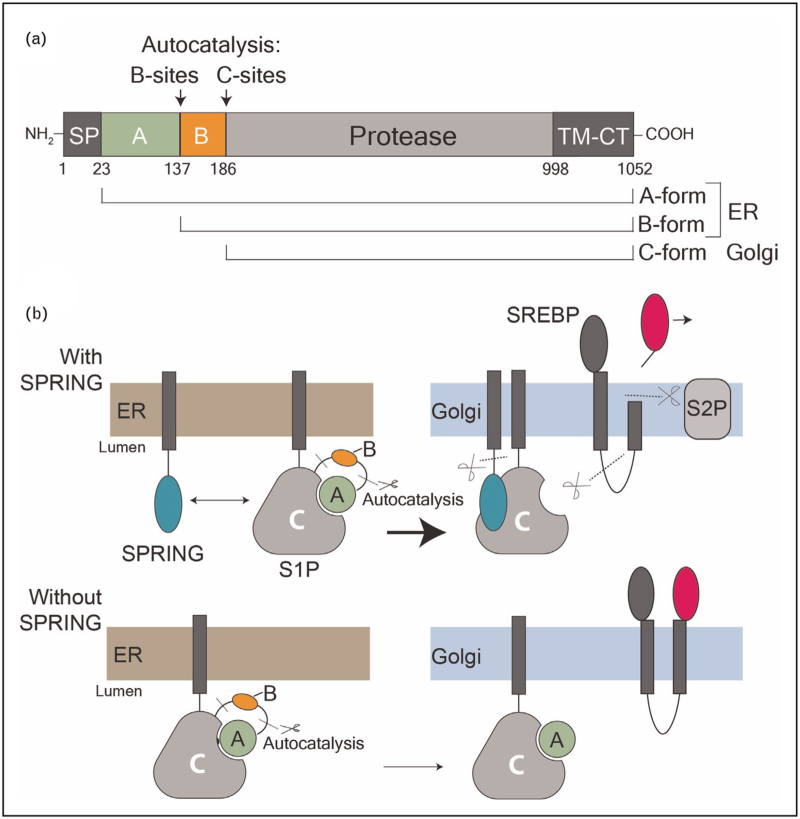
Architecture of S1P and functions of S1P-SPRING. (a) Schematic for the domains of S1P and its maturation. (b) Schematic for role of SPRING in accelerating the auto-maturation of S1P and licensing cleavage of SREBP in the Golgi. SPRING binding dissociates the inhibitory pro-domain of S1P. Signal peptide, SP; A-pro-domain, A; B-pro-domain, B; TM-CT, transmembrane-cytosolic tail.

S1P, the first SREBP-cleaving protease, was discovered in parallel by the Brown and Goldstein laboratory using a cDNA screen based on the cleavage of an SREBP2-based reporter [8], and by Seidah *et al*. [10], who cloned S1P (referred by them as SKI-1) by homology to other secretory pathway proteases. S1P belongs to the mammalian proprotein convertase family (PCSK), together with eight other serine proteases [11]. Like all members of this family, S1P is produced as a zymogen in the ER lumen. Once the catalytically competent state is reached, it undergoes two successive autocatalytic steps to cleave the A and B pro-domains, at the so-called B-sites and C-sites, respectively, thereby maturing into the active C-form (Fig. 2a) [12]. Using a whole-genome genetic screen, we recently discovered SREBF Regulating Gene (SPRING, formerly C12ORF49) as a novel key player in SREBPs signaling [13], a finding that was subsequently confirmed by other labs [14–16].

SPRING is a small type-II glycoprotein with a cysteine-rich C-terminal domain (CTD) that projects into the secretory lumen [13]. SPRING appears to be largely localized to the Golgi, although localization experiments have relied on over-expression experiments due to the lack of quality antibodies. Cell culture studies have established three biochemical functions for SPRING. First, SPRING and S1P form a complex and SPRING stimulates the maturation of S1P from its A-form to its C-form [15,17^▪▪^]. Second, SPRING specifically licenses S1P to cleave SREBPs [15,16,17^▪▪^,^18^^▪▪^] but, importantly, not other S1P substrates [17^▪▪^]. In the absence of SPRING, proteolytic maturation of SREBPs and SREBP-dependent signaling is dramatically abrogated. Importantly, SPRING is required for S1P to cleave SREBPs even when Golgi-localized S1P is returned to the ER through treatment with brefeldin A, which collapses the Golgi into ER [15,16]. This demonstrates that SPRING is required for S1P activity independent of its role in trafficking. Third, SPRING possesses a functional S1P cleavage motif (R_45_NLL_49_↓) between its N-terminal transmembrane helix and CTD [17^▪▪^]. The presence of SPRING peptides in human plasma samples strongly suggests this cleavage happens in humans [19–21], but the physiological significance of this event remains unclear (Fig. 2b). In this review, we summarize recent advances in understanding the mechanistic, structural, and physiologic role of the S1P-SPRING axis in SREBP signaling and highlight gaps that deserve further attention.

## THE PHYSIOLOGICAL ROLE OF SPRING

The *SPRING* transcript is ubiquitously expressed [13]. As with the global ablation of other core components of the SREBP pathway (Scap [22], SREBP1 [23], SREBP2 [24], and S1P [25]), the deletion of *Spring* in mice results in early embryonic lethality [13]. Even inducible global deletion of *Spring* in adult mice led to a rapid deterioration of their well being and to reaching humane endpoints, highlighting the central role of SPRING in SREBP signaling *in vivo*[26^▪▪^]. Given that SREBP-regulated DNL is a prominent process in the liver, we recently developed liver-specific *Spring* knockout (LKO) mice [26^▪▪^]. LKO mice are viable and show no overt phenotype. Absence of hepatic SPRING results in a drastic attenuation of SREBP signaling in LKO mice. In the liver, this is reflected by reduced proteolytic cleavage of SREBP1 and SREBP2, decreased expression of SREBP target-genes and their encoded proteins, diminished hepatic cholesterol and fatty acid content and biosynthesis, and attenuated hepatic VLDL secretion. Consequently, plasma cholesterol is dramatically decreased in all lipoprotein fractions. These effects were sex-independent, as they were observed in both male and female mice. Intriguingly, despite lower hepatic fatty acid synthesis and VLDL secretion, circulating triglyceride levels were unaffected. These observations cement the in-vivo role of SPRING in SREBP signaling [26^▪▪^].

The availability of the LKO mice also allowed evaluation of two important aspects of SPRING function. First, we investigated whether the cleavage of SPRING by S1P is necessary for SREBP signaling. Experiments in LKO mice and cells show that SREBP signaling can be rescued by introducing wildtype SPRING, a noncleavable SPRING mutant (R45E), or simply the ectodomain of SPRING (i.e., the cleaved SPRING fragment) ([17^▪▪^] and unpublished observations). Second, we asked whether SPRING is required for other S1P-dependent pathways. Since S1P has multiple substrates [27], one would expect SPRING deficiency to have pleiotropic effects. For example, S1P is involved in lysosome biogenesis because it cleaves the precursor form of N-acetylglucosamine-1-phosphotransferase (encoded by *GNPTAB*) into its α and β subunits [28]. The matured GNPTα/β catalyzes the first step in the production of the mannose 6-phosphate (M6P) posttranslational modification that targets luminal proteins to the lysosome. Accordingly, Xiao *et al.*[15] implicated SPRING in lysosomal biogenesis in HeLa cells. In contrast, we have not observed changes in lysosome amount and size in cell models lacking SPRING (unpublished). Moreover, transcriptional and proteomic analysis of LKO livers and of SPRING-deficient cells revealed only alterations in the SREBP pathway, with no changes measured in the ATF6 and CREB3L3 signaling pathways that also require S1P for their activation ([17^▪▪^,^26^^▪▪^] and unpublished). It is interesting that Bayraktar *et al.* [16] reported that Zebrafish lacking *Spring* showed lipid absorption defects comparable to that of *mbtps1*-mutants. While we cannot formally rule out that in Zebrafish SPRING broadly governs S1P activity, this is not the case in LKO mice and in SPRING-deficient mammalian cell models. Rather, absence of SPRING more closely resembles Scap deficiency [29]. Interestingly, in our initial report, we identified an interaction between SPRING and Scap and suggested it may contribute to the retrograde transport of Scap from the Golgi to the ER, a necessary step in the SREBP cycle (Fig. 1) [13,30–32]. A connection between SREBPs, Scap, and SPRING is further supported by proximity ligation data [14], and rare human mutations to the S1P cleavage site in SREBP2 that produce a pleiotropic phenotype partially reminiscent of recessive S1P mutations [33^▪^,^34^]. Certainly, the significance of the SPRING-Scap interaction in the Golgi warrants further structural and functional investigation.

Our knowledge of the role of SPRING in human lipid metabolism is more limited. Analysis of the Global Lipids Genetics Consortium and the UK Biobank identified 17 Single Nucleotide Polymorphisms in *SPRING* that correlated with increased circulating levels of HDL-c [26^▪▪^]. The lead-associated variant, rs10507274-C, was also associated with reduced circulating LDL-c and triglycerides, albeit without reaching genome-wide statistical significance [26^▪▪^]. This variant produces a Q55R substitution, the functional significance of which remains unknown. Whether SPRING plays a role in human disease is not yet clear. A recent study demonstrates a correlation between *SPRING* expression and hepatocellular carcinoma (HCC) progression [35^▪^]. Higher *SPRING* expression correlated with enhanced tumor growth and poor prognosis, likely reflecting the tumoral demand for DNL to support proliferation. Reciprocally, silencing *SPRING* expression decreases proliferation of HCC tumor cells. It will be interesting to see whether *SPRING* expression emerges as a pan-cancer proliferation marker and potential target, or if the dependence on SPRING is HCC-specific.

## THE S1P-SPRING AXIS CONTROLS PROTEOLYTIC ACTIVATION OF SREBPS

**FIGURE 3:**
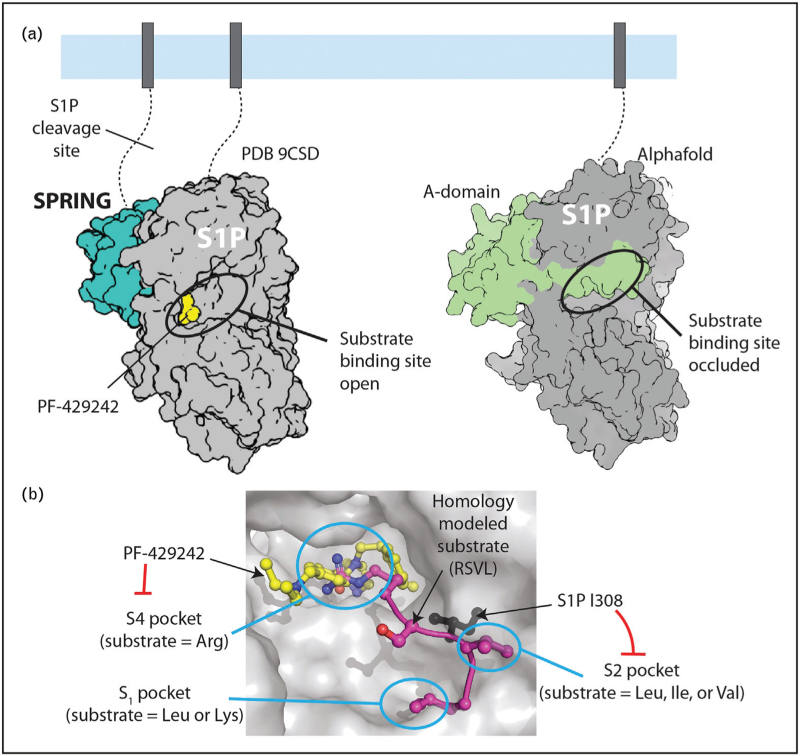
Structural basis for activation of S1P by SPRING. (a) Structural models of S1P with and without SPRING. *Left*: Model for the S1P-SPRING complex based on PDB 9CSD. The S1P competitive inhibitor, PF-429242 (yellow spheres), is bound in the S4 pocket of the active site. *Right*: Model for S1P without SPRING based on alphafold and PDB 8UWC. The S1P A-domain occupies the active site. S1P C-form ectodomain shown in grey. (b) Schematic for the substrate binding site of S1P, competitive inhibition by PF-429242, and role of I308 in controlling access to the S2 pocket (PDB 9CSD).

To gain a mechanistic understanding of how SPRING licenses S1P activity, we purified the enzymatic ectodomain of S1P alone and as a stable complex with SPRING. S1P purified without SPRING remained associated with its inhibitory pro-domain and was largely inactive, whereas S1P co-purified with SPRING was enzymatically active [18^▪▪^]. This interaction between the matured C-form S1P and its pro-domain was consistent with previous reports (Fig. 2b) [12,36,37]. Structures of S1P ± SPRING obtained using cryo-electron microscopy (cryo-EM) revealed that the cleaved A domain remains tightly associated with the protease domain of S1P, occluding the active site, and trapping the substrate-binding pocket in a conformation that is refractory to substrate binding. In contrast, SPRING displaces the inhibitory A-domain, makes the active site available for substrates, and stabilizes interactions between subdomains of S1P to position key residues involved in catalysis (Fig. 3a) [18^▪▪^].

Studies in cells have shown that SPRING accelerates the transition of S1P from the B-form to the C-form [15,17^▪▪^]. Xiao *et al.* [15] proposed that SPRING directs an alternate order of cleavage events, causing S1P to skip cleavage at its B-site and instead cleave directly at the C-site. Because the B-site motif occupies the S1P active site in the absence of SPRING, we proposed that SPRING accelerates S1P maturation to the C-form by liberating the active site from the B-site motif and enabling cleavage at the C-site, which may be taken as a molecular mark of a fully activated S1P [18^▪▪^]. This interpretation is consistent with mutagenesis data indicating that mutation of the B-site abolishes S1P maturation and that cleavage at the C-site is not required for most S1P activities [12,17^▪▪^,^36^].

## RATIONALE FOR INHIBITORS OF S1P/SPRING

S1P cleaves substrates with the recognition sequence R_4_X_3_(L/I/V)_2_Z_1_↓, where X is not cysteine or proline, and Z is preferably leucine or lysine. This substrate specificity is unique within the PCSK family [11]. In this parlance, P# represents the substrate peptide residue, numbered sequentially from the cleavage site (↓). The P# residue binds in the corresponding S# pocket on the protease. For example, the P_4_ Arg from a substrate binds in the S_4_ pocket of S1P (Fig. 3b). The potential for specific competitive inhibition of S1P was demonstrated with the small molecule PF-429242 that has become widely used in cell culture studies [38,39]. Structural analysis of S1P/SPRING bound with PF-429242 showed that PF-429242 binds in the acidic S_4_ pocket where it competes with the P_4_ Arg residue of the substrate (Fig. 3a,b) [40^▪▪^]. Crucially, the S_4_ pocket is preformed; that is, it was observed in the structure of S1P/SPRING without any inhibitor. In contrast, the predicted S_2_ pocket for the hydrophobic P_2_ (L/I/V) residue was sterically occluded by the S1P residue I308. Substrate binding by S1P presumably includes an as-of-yet unobserved conformation change to open the S_2_ pocket to accommodate the hydrophobic P_2_ side chain [40^▪▪^]. This steric block of the P_2_ pocket in apo S1P/SPRING likely potentiates the efficacy of PF-429242 as introducing a mutation to open the pocket (I308A) increased S1P/SPRING activity and reduced the efficacy of PF-429242.

Targeting the interaction between SPRING and S1P may offer another route for specific inhibition of S1P in two interesting ways. First, as described above, genetic ablation of *SPRING* specifically disrupts SREBP processing while leaving other S1P-dependent pathways intact [13,17^▪▪^,^26^^▪▪^]. In contrast, PF-429242 has been used to inhibit non-SREBP activities of S1P [41–44]. Second, SPRING has a unique structure and its binding site on S1P is unique within the PCSK family [18^▪▪^]. Therefore, molecules that disrupt this protein-protein interaction may offer specificity for S1P/SPRING.

## OPEN QUESTIONS REGARDING THE S1P-SPRING AXIS

The text above summarizes our current mechanistic and physiologic understanding of the S1P-SPRING axis in controlling SREBP-regulated lipid metabolism, since its discovery in 2020 [13]. The studies covered in this review highlight the role of SPRING in the SREBP pathway, and position it, next to Scap, S1P, S2P, and INSIGs [4], as a core component of SREBP activation machinery (Fig. 1). Yet these studies also reveal several gaps whose elucidation we consider important.

### How are SREBPs licensed as S1P substrates by SPRING?

The substrate specificity of S1P includes a wide variety of proteins harboring the canonical RXXL cleavage motif [27]. How SPRING enables S1P to discriminate between SREBPs and its other substrates is not known. A notable distinction between SREBPs and other S1P substrates is that the former are delivered to the Golgi in complex with their dedicated chaperone Scap. We have shown earlier that SPRING interacts with Scap [13]. SPRING may specifically support the proteolytic cleavage of SREBPs by S1P by enabling the interaction of S1P with an incoming Scap-SREBP complex.

### What is the function of SPRING cleavage?

SPRING contains a canonical S1P cleavage motif (RNLL_49_↓) [13] that is functional in cells and mice [17^▪▪^] and likely occurs in humans [19–21]. However, we have reported that S1P-mediated SPRING cleavage is dispensable for gross activation of SREBP signaling, and at present the significance of this event is unclear [17^▪▪^]. Possibly, cleavage of SPRING (and S1P shedding [45]) may serve to integrate specific as-of-yet unidentified metabolic cues to fine-tune SREBP activation.

### Do other S1P family members have “SPRING-like” chaperones?

Currently, two of the nine PCSKs are known to require a chaperone for activity. In addition to S1P and SPRING, PCSK2 is stabilized for activity by its chaperone protein 7B2 (reviewed in [46]). Whether 7B2 functions similarly to SPRING and displaces the pro-domain of PCSK2 remains unknown and will require determining the structure of that complex. Given their similar mechanism of auto-proteolytic activation and the need to control the cellular site of activity, it is tempting to speculate that the other PCSKs have chaperones awaiting discovery.

### Can the S1P-SPRING axis be targeted in therapeutic strategies to treat cardio-metabolic disease?

Based on the SREBP-specific role of SPRING, targeting the SPRING-S1P complex should circumvent the pleiotropic effects of pan-S1P inhibition. In addition to the two strategies described above, inhibiting the S1P-SPRING complex can be achieved by silencing *SPRING* expression. The unique specificity of the S1P-SPRING axis for SREBP activation makes this complex an attractive target for limiting *DNL* in, most notably, cardio-metabolic disease and malignancies.

## CONCLUSION

In the 5 years since its discovery, SPRING has emerged as a component of the core SREBP machinery responsible for the sterol-regulated maturation of those transcription factors. Further elucidation of the connections between SPRING and the SREBP pathway will increase our understanding of lipid metabolism and provide new avenues for the rationale design of molecular therapies to treat metabolic dysfunction.

## Acknowledgements

*None.*

### Financial support and sponsorship

*The study of S1P and SPRING in our laboratories in supported by the National Institutes of Health (R00GM141261 and R01GM155152) and the Welch Foundation (I-2246–20250403) (to D.L.K.). N.Z. is an Established Investigator of the Dutch Heart Foundation (2013T111) and is supported by a Vici grant from the Netherlands Organization for Scientific Research (NWO; 016.176.643) and an NWO ENW grant (M.22.034; GENESIS). The authors thank Sebastian Hendrix for assisting with graphics, Irith Koster and the members of the Zelcer and Kober laboratories for their critical comments and suggestions on this study.*

### Conflicts of interest

*The authors declare they have no conflict of interest.*

## REFERENCES AND RECOMMENDED READING

Papers of particular interest, published within the annual period of review, have been highlighted as:

▪ of special interest
▪▪ of outstanding interest

## References

[1] Goldstein JL DeBose-Boyd RA Brown MS. Protein sensors for membrane sterols. Cell 2006; 124:35–46. 16413480 10.1016/j.cell.2005.12.022

[2] Hua X Yokoyama C Wu J. SREBP-2, a second basic-helix-loop-helix-leucine zipper protein that stimulates transcription by binding to a sterol regulatory element. Proc Natl Acad Sci U S A 1993; 90:11603–11607. 7903453 10.1073/pnas.90.24.11603 PMC48032

[3] Yokoyama C Wang X Briggs MR. SREBP-1, a basic-helix-loop-helix-leucine zipper protein that controls transcription of the low density lipoprotein receptor gene. Cell 1993; 75:187–197. 8402897

[4] Brown MS Radhakrishnan A Goldstein JL. Retrospective on cholesterol homeostasis: the central role of scap. Annu Rev Biochem 2018; 87:783–807. 28841344 10.1146/annurev-biochem-062917-011852 PMC5828883

[5] Sakai J Duncan EA Rawson RB. Sterol-regulated release of SREBP-2 from cell membranes requires two sequential cleavages, one within a transmembrane segment. Cell 1996; 85:1037–1046. 8674110 10.1016/s0092-8674(00)81304-5

[6] Radhakrishnan A Goldstein JL McDonald JG Brown MS. Switch-like control of SREBP-2 transport triggered by small changes in ER cholesterol: a delicate balance. Cell Metab 2008; 8:512–521. 19041766 10.1016/j.cmet.2008.10.008 PMC2652870

[7] Kober DL Radhakrishnan A Goldstein JL. Scap structures highlight key role for rotation of intertwined luminal loops in cholesterol sensing. Cell 2021; 184:3689–3701. e3622. 34139175 10.1016/j.cell.2021.05.019 PMC8277531

[8] Sakai J Rawson RB Espenshade PJ. Molecular identification of the sterol-regulated luminal protease that cleaves SREBPs and controls lipid composition of animal cells. Mol Cell 1998; 2:505–514. 9809072 10.1016/s1097-2765(00)80150-1

[9] Rawson RB Zelenski NG Nijhawan D. Complementation cloning of S2P, a gene encoding a putative metalloprotease required for intramembrane cleavage of SREBPs. Mol Cell 1997; 1:47–57. 9659902 10.1016/s1097-2765(00)80006-4

[10] Seidah NG Mowla SJ Hamelin J. Mammalian subtilisin/kexin isozyme SKI-1: a widely expressed proprotein convertase with a unique cleavage specificity and cellular localization. Proc Natl Acad Sci U S A 1999; 96:1321–1326. 9990022 10.1073/pnas.96.4.1321 PMC15461

[11] Seidah NG Prat A. The biology and therapeutic targeting of the proprotein convertases. Nat Rev Drug Discov 2012; 11:367–383. 22679642 10.1038/nrd3699

[12] Espenshade PJ Cheng D Goldstein JL Brown MS. Autocatalytic processing of site-1 protease removes propeptide and permits cleavage of sterol regulatory element-binding proteins. J Biol Chem 1999; 274:22795–22804. 10428864 10.1074/jbc.274.32.22795

[13] Loregger A Raaben M Nieuwenhuis J. Haploid genetic screens identify SPRING/C12ORF49 as a determinant of SREBP signaling and cholesterol metabolism. Nat Commun 2020; 11:1128. 32111832 10.1038/s41467-020-14811-1 PMC7048761

[14] Aregger M Lawson KA Billmann M. Systematic mapping of genetic interactions for de novo fatty acid synthesis identifies C12orf49 as a regulator of lipid metabolism. Nat Metab 2020; 2:499–513. 32694731 10.1038/s42255-020-0211-z PMC7566881

[15] Xiao J Xiong Y Yang LT. POST1/C12ORF49 regulates the SREBP pathway by promoting site-1 protease maturation. Protein Cell 2021; 12:279–296. 32666500 10.1007/s13238-020-00753-3 PMC8019017

[16] Bayraktar EC La K Karpman K. Metabolic coessentiality mapping identifies C12orf49 as a regulator of SREBP processing and cholesterol metabolism. Nat Metab 2020; 2:487–498. 32694732 10.1038/s42255-020-0206-9 PMC7384252

[17▪▪] Hendrix S Tan JME Ndoj K. SPRING is a dedicated licensing factor for SREBP-specific activation by S1P. Mol Cell Biol 2024; 44:123–137. 38747374 10.1080/10985549.2024.2348711 PMC11110692

[18▪▪] Hendrix S Dartigue V Hall H. SPRING licenses S1P-mediated cleavage of SREBP2 by displacing an inhibitory pro-domain. Nat Commun 2024; 15:5732. 38977690 10.1038/s41467-024-50068-8 PMC11231238

[19] Keshishian H Burgess MW Gillette MA. Multiplexed, quantitative workflow for sensitive biomarker discovery in plasma yields novel candidates for early myocardial injury. Mol Cell Proteomics 2015; 14:2375–2393. 25724909 10.1074/mcp.M114.046813 PMC4563722

[20] Dey KK Wang H Niu M. Deep undepleted human serum proteome profiling toward biomarker discovery for Alzheimer's disease. Clin Proteomics 2019; 16:16. 31019427 10.1186/s12014-019-9237-1 PMC6472024

[21] Pernemalm M Sandberg A Zhu Y. In-depth human plasma proteome analysis captures tissue proteins and transfer of protein variants across the placenta. Elife 2019; 8:e41608. 30958262 10.7554/eLife.41608 PMC6519984

[22] Matsuda M Korn BS Hammer RE. SREBP cleavage-activating protein (SCAP) is required for increased lipid synthesis in liver induced by cholesterol deprivation and insulin elevation. Genes Dev 2001; 15:1206–1216. 11358865 10.1101/gad.891301 PMC313801

[23] Shimano H Shimomura I Hammer RE. Elevated levels of SREBP-2 and cholesterol synthesis in livers of mice homozygous for a targeted disruption of the SREBP-1 gene. J Clin Invest 1997; 100:2115–2124. 9329978 10.1172/JCI119746 PMC508404

[24] Vergnes L Chin RG de Aguiar Vallim T. SREBP-2-deficient and hypomorphic mice reveal roles for SREBP-2 in embryonic development and SREBP-1c expression. J Lipid Res 2016; 57:410–421. 26685326 10.1194/jlr.M064022 PMC4766990

[25] Yang J Goldstein JL Hammer RE. Decreased lipid synthesis in livers of mice with disrupted Site-1 protease gene. Proc Natl Acad Sci U S A 2001; 98:13607–13612. 11717426 10.1073/pnas.201524598 PMC61088

[26▪▪] Hendrix S Kingma J Ottenhoff R. Hepatic SREBP signaling requires SPRING to govern systemic lipid metabolism in mice and humans. Nat Commun 2023; 14:5181. 37626055 10.1038/s41467-023-40943-1 PMC10457316

[27] Danyukova T Schoneck K Pohl S. Site-1 and site-2 proteases: a team of two in regulated proteolysis. Biochim Biophys Acta Mol Cell Res 2022; 1869:119138. 34619164 10.1016/j.bbamcr.2021.119138

[28] Marschner K Kollmann K Schweizer M. A key enzyme in the biogenesis of lysosomes is a protease that regulates cholesterol metabolism. Science 2011; 333:87–90. 21719679 10.1126/science.1205677

[29] Moon YA Liang G Xie X. The Scap/SREBP pathway is essential for developing diabetic fatty liver and carbohydrate-induced hypertriglyceridemia in animals. Cell Metab 2012; 15:240–246. 22326225 10.1016/j.cmet.2011.12.017 PMC3662050

[30] Kober DL Xu S Li S. Identification of a degradation signal at the carboxy terminus of SREBP2: a new role for this domain in cholesterol homeostasis. Proc Natl Acad Sci U S A 2020; 117:28080–28091. 33106423 10.1073/pnas.2018578117 PMC7668084

[31] Takashima K Saitoh A Funabashi T. COPI-mediated retrieval of SCAP is crucial for regulating lipogenesis under basal and sterol-deficient conditions. J Cell Sci 2015; 128:2805–2815. 26092941 10.1242/jcs.164137

[32] Nohturfft A DeBose-Boyd RA Scheek S. Sterols regulate cycling of SREBP cleavage-activating protein (SCAP) between endoplasmic reticulum and Golgi. Proc Natl Acad Sci U S A 1999; 96:11235–11240. 10500160 10.1073/pnas.96.20.11235 PMC18017

[33▪] Moulton MJ Atala K Zheng Y. Dominant missense variants in SREBF2 are associated with complex dermatological, neurological, and skeletal abnormalities. Genet Med 2024; 26:101174. 38847193 10.1016/j.gim.2024.101174 PMC12358889

[34] Kondo Y Fu J Wang H. Site-1 protease deficiency causes human skeletal dysplasia due to defective inter-organelle protein trafficking. JCI Insight 2018; 3:121596. 30046013 10.1172/jci.insight.121596 PMC6124414

[35▪] Yu HC Jin L Bai L. C12ORF49 inhibits ferroptosis in hepatocellular carcinoma cells via reprogramming SREBP1/SCD1-mediated lipid metabolism. Cell Death Discov 2025; 11:178. 40240331 10.1038/s41420-025-02480-2 PMC12003882

[36] da Palma JR Burri DJ Oppliger J. Zymogen activation and subcellular activity of subtilisin kexin isozyme 1/site 1 protease. J Biol Chem 2014; 289:35743–35756. 25378398 10.1074/jbc.M114.588525 PMC4276844

[37] Toure BB Munzer JS Basak A. Biosynthesis and enzymatic characterization of human SKI-1/S1P and the processing of its inhibitory prosegment. J Biol Chem 2000; 275:2349–2358. 10644685 10.1074/jbc.275.4.2349

[38] Hawkins JL Robbins MD Warren LC. Pharmacologic inhibition of site 1 protease activity inhibits sterol regulatory element-binding protein processing and reduces lipogenic enzyme gene expression and lipid synthesis in cultured cells and experimental animals. J Pharmacol Exp Ther 2008; 326:801–808. 18577702 10.1124/jpet.108.139626

[39] Hay BA Abrams B Zumbrunn AY. Aminopyrrolidineamide inhibitors of site-1 protease. Bioorg Med Chem Lett 2007; 17:4411–4414. 17583500 10.1016/j.bmcl.2007.06.031

[40▪▪] Bullington AV Micallo I Bajaj B. Structural basis for substrate selectivity by site-one protease revealed by studies with a small-molecule inhibitor. Proc Natl Acad Sci U S A 2025; 122:e2426931122. 40299693 10.1073/pnas.2426931122 PMC12067269

[41] Chen X Zhang J Liu P. Proteolytic processing of secretory pathway kinase Fam20C by site-1 protease promotes biomineralization. Proc Natl Acad Sci U S A 2021; 118:e2100133118. 34349020 10.1073/pnas.2100133118 PMC8364200

[42] Pasquato A Rochat C Burri DJ. Evaluation of the antiarenaviral activity of the subtilisin kexin isozyme-1/site-1 protease inhibitor PF-429242. Virology 2012; 423:14–22. 22154237 10.1016/j.virol.2011.11.008 PMC3285533

[43] Gallagher CM Garri C Cain EL. Ceapins are a new class of unfolded protein response inhibitors, selectively targeting the ATF6alpha branch. Elife 2016; 5: 10.7554/eLife.11878 PMC4954757 27435960

[44] Pechincha C Groessl S Kalis R. Lysosomal enzyme trafficking factor LYSET enables nutritional usage of extracellular proteins. Science 2022; 378:eabn5637. 36074822 10.1126/science.abn5637

[45] Elagoz A Benjannet S Mammarbassi A. Biosynthesis and cellular trafficking of the convertase SKI-1/S1P: ectodomain shedding requires SKI-1 activity. J Biol Chem 2002; 277:11265–11275. 11756446 10.1074/jbc.M109011200

[46] Mbikay M Seidah NG Chretien M. Neuroendocrine secretory protein 7B2: structure, expression and functions. Biochem J 2001; 357:329–342. 11439082 10.1042/0264-6021:3570329 PMC1221959

